# The impact of the first year of COVID-19 pandemic on suicides in a collection of 27 EU-related countries

**DOI:** 10.1038/s41598-024-68604-3

**Published:** 2024-07-30

**Authors:** Tamás Lantos, Tibor András Nyári

**Affiliations:** Department of Medical Physics and Informatics, Albert Szent-Györgyi Medical School, University of Szeged, 9 Korányi Alley, Szeged, 6720 Hungary

**Keywords:** Public health, Risk factors

## Abstract

Disasters, including epidemics, have a characteristic course, both in terms of the specific events and the human reactions to them. However, it is difficult to predict whether the COVID-19 pandemic will eventually lead to an increase in suicide rates. We aimed to provide a general pattern of the change in suicide rates in the countries linked to the European Union by direct comparison of the years 2019 and 2020 by gender and age group, grouped according to the predominant religions. Overall, 27 countries were included in the analysis. Incidence rate ratios and their 95% confidence intervals were calculated to characterise annual changes in the incidence of suicide deaths. In almost two-thirds of the countries studied, suicide rates did not increase. The largest increases were observed in Catholic-majority and ‘mixed’ Catholic-Protestant countries, but this was significant only for the oldest age group (over 65 years). This increase was even more marked within some Catholic-majority countries (Hungary, Ireland, and Spain) during the first months of the pandemic. There was no statistically significant increase overall in the suicide death rates in Europe. However, the pattern of suicide rates has changed significantly in some countries, and by age group and religion, respectively.

## Introduction

Epidemics are considered disasters both psychologically and legally. An important attribute of a disaster is that it has a characteristic course, both in terms of the specific events and the human reactions to them^1^.

However, the outbreak in 2020 affected the whole world (the World Health Organisation declared it a pandemic on 11 March) and almost simultaneously: there were no ‘protected zones’. It should be mentioned that no disaster has yet hit people so that personal (‘face-to-face’) contact has had to be completely prohibited; only 21st-century IT technology could help—but exclusively for those who had such devices and the skills needed to manage them^2^.

Both confinement and isolation are serious sources of stress^3^ (even separately): family conflicts (and abuse as its extreme form) have increased as a destructive consequence of confinement, and isolation has led to loneliness associated with complete hopelessness for many older and/or single people.

In addition to making contact (more) difficult, the quarantine period also prevented the maintenance of many traditional ritual gatherings (liturgies, funerals, weddings). The closure/shutdown of workplaces further exacerbated the deterioration of mental (and often even somatic) health.

The social distancing and quarantine imposed during the first wave of the pandemic meant that the most vulnerable groups (i.e., the elderly and the chronically ill) needed social support (purchasing food and medicines). Many volunteers participated in this, working together in an organised way. Similar help (housing, childcare) was needed for ‘frontline’ health workers^2^ as they were exposed to suicidal thoughts and burnout^4^.

The increased workload in the entire healthcare system due to the COVID-19 pandemic impairs access to mental health services for chronic psychiatric patients, which can also lead to a worsening of their condition. However, since the pandemic ‘triggers’ protective mechanisms (social cohesion, ‘pulling together’ phenomenon) in addition to the numerous risk factors, it is difficult to predict whether this will eventually lead to an increase in suicide rates^5^.

This question has been investigated in several countries at the population level worldwide^6–18^ and specifically in Europe^5,19–24^, using a wide variety of statistical methods^25^. Although overall (and in most cases) no increase was detected, the picture was very mixed. It is worth mentioning the case of the province of Lleida (Lérida) located in Catalonia, Spain, where the number of suicide deaths almost halved from 2019 to 2020^26^.

Suicide is a multifactorial phenomenon; the chance of its occurrence is determined by several factors. These include gender^27^, age^28^ and religion^29^: risk– and protective factors (and thus suicide rates) vary along these subgroups. In our present study, we aimed to provide a general pattern of the change in suicidal rates in countries (directly and indirectly) linked to the European Union by direct comparison of the years 2019 and 2020 by gender and age group, grouped according to the predominant religions.

## Results

### Maps at a glance

**Figure 1:**
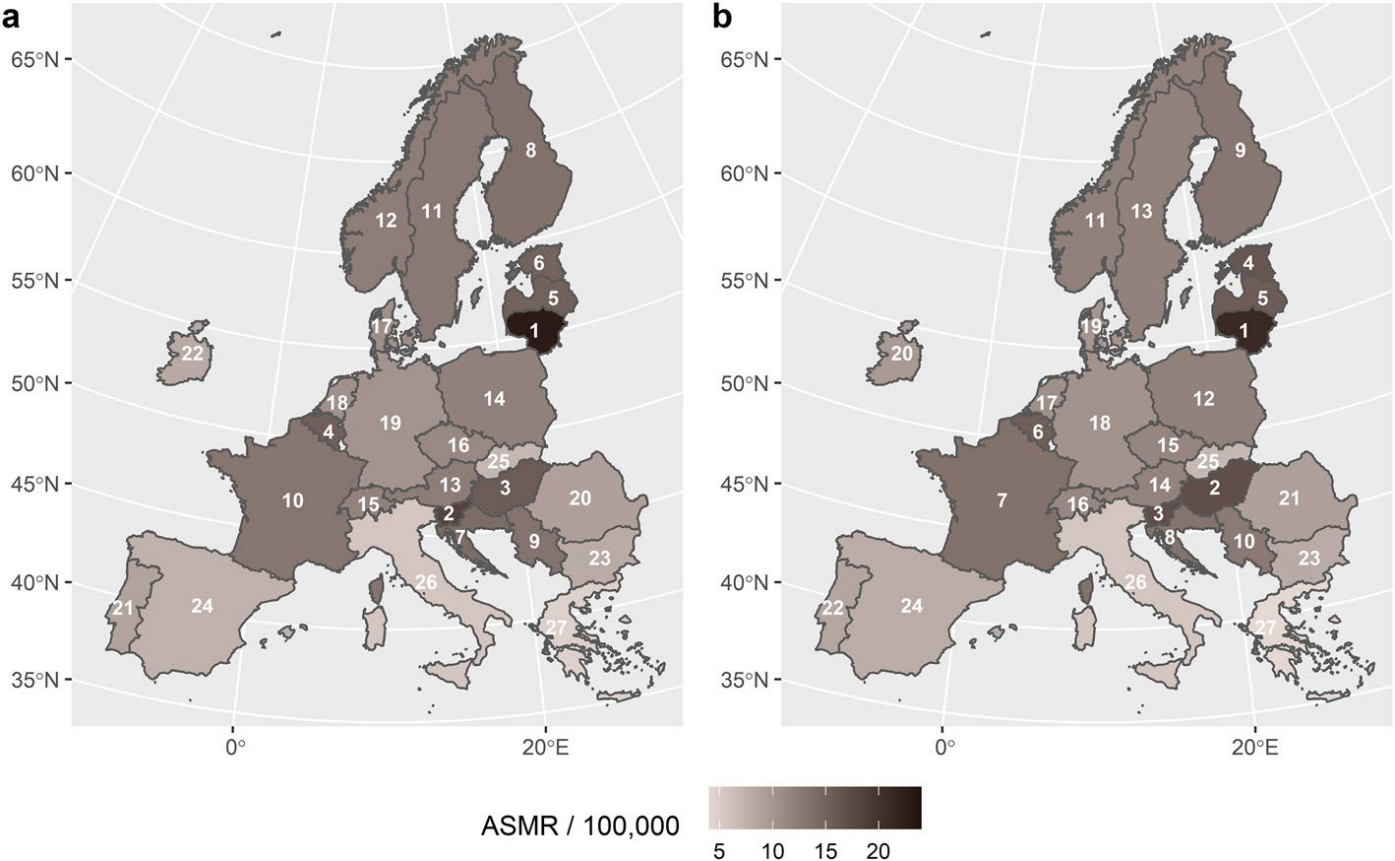
The 27 countries under investigation coloured and labelled (rank) by suicide rates. (**a)** In 2019. (**b)** In 2020. Age-standardised mortality rates (ASMR) were expressed per 100,000 capita. The maps were created using R, packages *sf* (version 1.0–12; https://cran.r-project.org/web/packages/sf/index.html) and *ggplot2* (version 3.3.5; https://cran.r-project.org/web/packages/ggplot2/index.html).

Figure 1 shows a map display of the 27 examined countries, coloured by their standardised suicide rates (directly obtained from Eurostat's *Data Browser*) and labelled by their relative position (rank), all placed side by side in years 2019 (Fig. 1a) and 2020 (Fig. 1b).

The overall picture of the two maps is very similar: the highest rates are in the Baltic states (Estonia, Latvia, and Lithuania), Hungary, Slovenia, and Belgium; the lowest rates are in the three most populous southern countries (Italy, Spain, and Greece) and Slovakia.

### *Meta*-analytic approach

However, the relative change (incidence rate ratio; IRR) and the corresponding 95% confidence interval (95% CI) for each country describe a more relevant pattern in the European suicide mortality rate.

**Figure 2:**
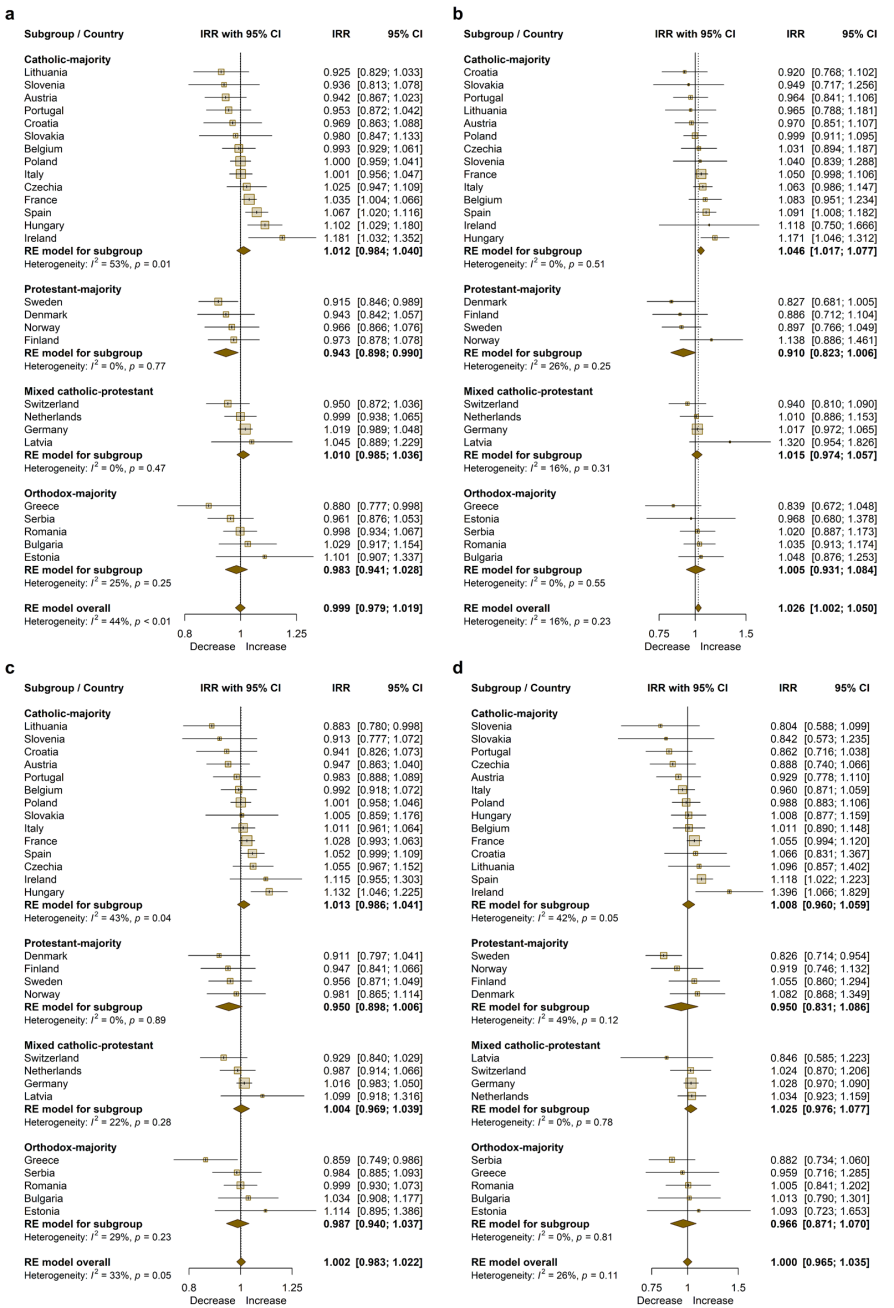
Forest plots for subgroup analyses of annual changes in the incidence of suicide deaths. (**a)** Total (whole) population. (**b)** Subpopulation aged over 65 years (**c)** Male subpopulation (**d)** Female subpopulation. The plots were generated using the R *meta* package (version 6.1–0; https://cran.r-project.org/web/packages/meta/index.html). *Notes*: *IRR* Incidence Rate Ratio; *CI* Confidence Interval; *RE* Random-Effects.

In Fig. 2, IRRs were displayed on forest plots for the total (whole) population (Fig. 2a), for the subpopulation over 65 years of age (as the pooled effect size was not significant for the other age groups) (Fig. 2b), and for the male (Fig. 2c) and female subpopulations (Fig. 2d), respectively.

It can be seen (Fig. 2a) that significant increases were only observed in Ireland (18.1%), Hungary (10.2%), Spain (6.7%) and France (3.5%); however, the increases in Estonia (10.1%) and Latvia (4.5%) were not statistically significant due to the smaller population size.

For those aged 65 years and older (Fig. 2b), the pooled effect size was significant (IRR = 1.026; 95% CI 1.002–1.050), but significant increases were only observed in Hungary (17.1%) and Spain (9.1%).

For males (Fig. 2c), a significant increase was observed only in Hungary (13.2%); for females (Fig. 2d), significant increases were found in Ireland (39.6%) and Spain (11.8%).

Regarding the breakdown by religion, the largest increases were observed in Catholic-majority and ‘mixed’ Catholic-Protestant countries (even decreases could be seen in Protestant-majority countries), but this was significant only for the group aged over 65 years, and only in Catholic-majority countries (IRR = 1.046; 95% CI 1.017–1.077). Considering this age group by gender (see Supplementary Fig. S1 online), the same can be observed for males: the increase was significant but only in the subgroup for Catholic-majority countries (IRR = 1.050; 95% CI 1.016–1.085). However, the increase was not significant for females: either overall (in this age group) or in the subgroup for Catholic-majority countries (IRR = 1.029; 95% CI 0.970–1.091).

We also performed sensitivity (leave-one-out) analyses, excluding Ireland and/or Hungary from the analysis in those cases where they ‘stand out’—to see how much the situation would change in terms of pooled effect size and heterogeneity within the subgroup.

If we left out Ireland and then Hungary from the analysis for total populations (Fig. 2a), Hungary for male subpopulations (Fig. 2c) and Ireland for female subpopulations (Fig. 2d), the heterogeneity did not fall below the threshold (*p* = 0.1) for any subgroup, while the pooled effect size (in the subgroup and overall) did not change substantially.

### Joinpoint regression: turning points and reversed trends

The six countries mentioned above (Ireland, Hungary, Estonia, Spain, Latvia, and France) deserve to be examined separately: whether the ‘relevant’ rises (in terms of their volume or even in statistical terms) were decisive in reversing the previous (potential) downward trend.

Essentially, there are two possible scenarios: either (1) the increase experienced in 2020 ‘triggers’ an upward phase (which starts at a so-called turning point in a certain year and ends in 2020) that was not observed earlier (up to and including 2019), maybe an already ascending trend turns (even statistically) significant, or (2) it does not.

**Figure 3:**
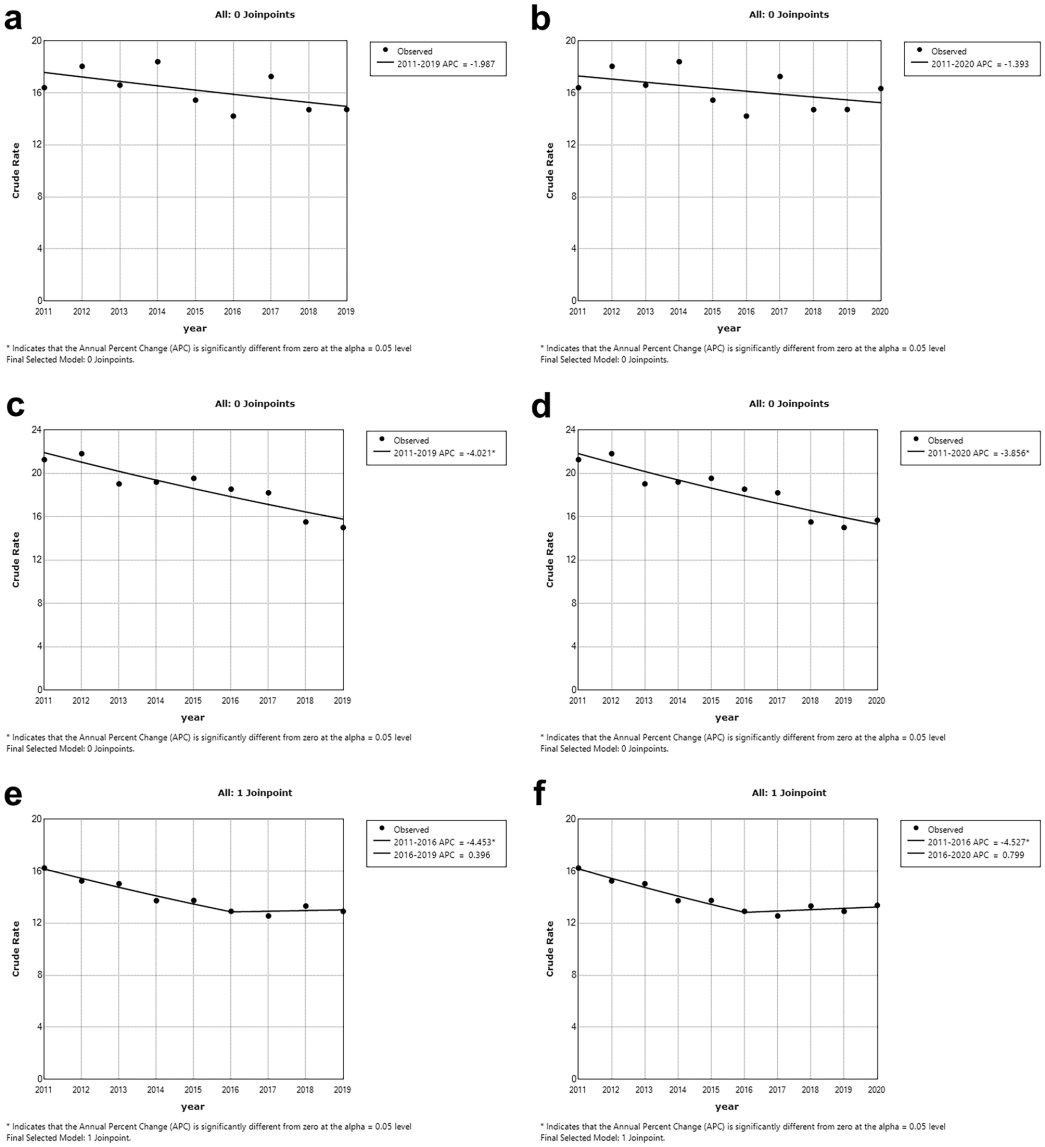
Trends in suicide death rates for countries with the highest (percentage) increase in 2020 using JP regression for the periods 2011–2019 (left side) and 2011–2020 (right side). (**a,b)** Estonia (**c,d)** Latvia (**e,f)** France. *Notes*: *JP* Joinpoint; *APC* Annual Percent Change.

Figure 3 shows the countries (period 2011–2019 on the left and 2011–2020 on the right, respectively), where the trend has remained (basically) unchanged: in the case of Estonia (Fig. 3a,b) and Latvia (Fig. 3c,d), a downward trend has been slightly reduced (but not ‘reversed’), while in the case of France (Fig. 3e,f), an already increasing trend has intensified (but still has not become significant).

**Figure 4:**
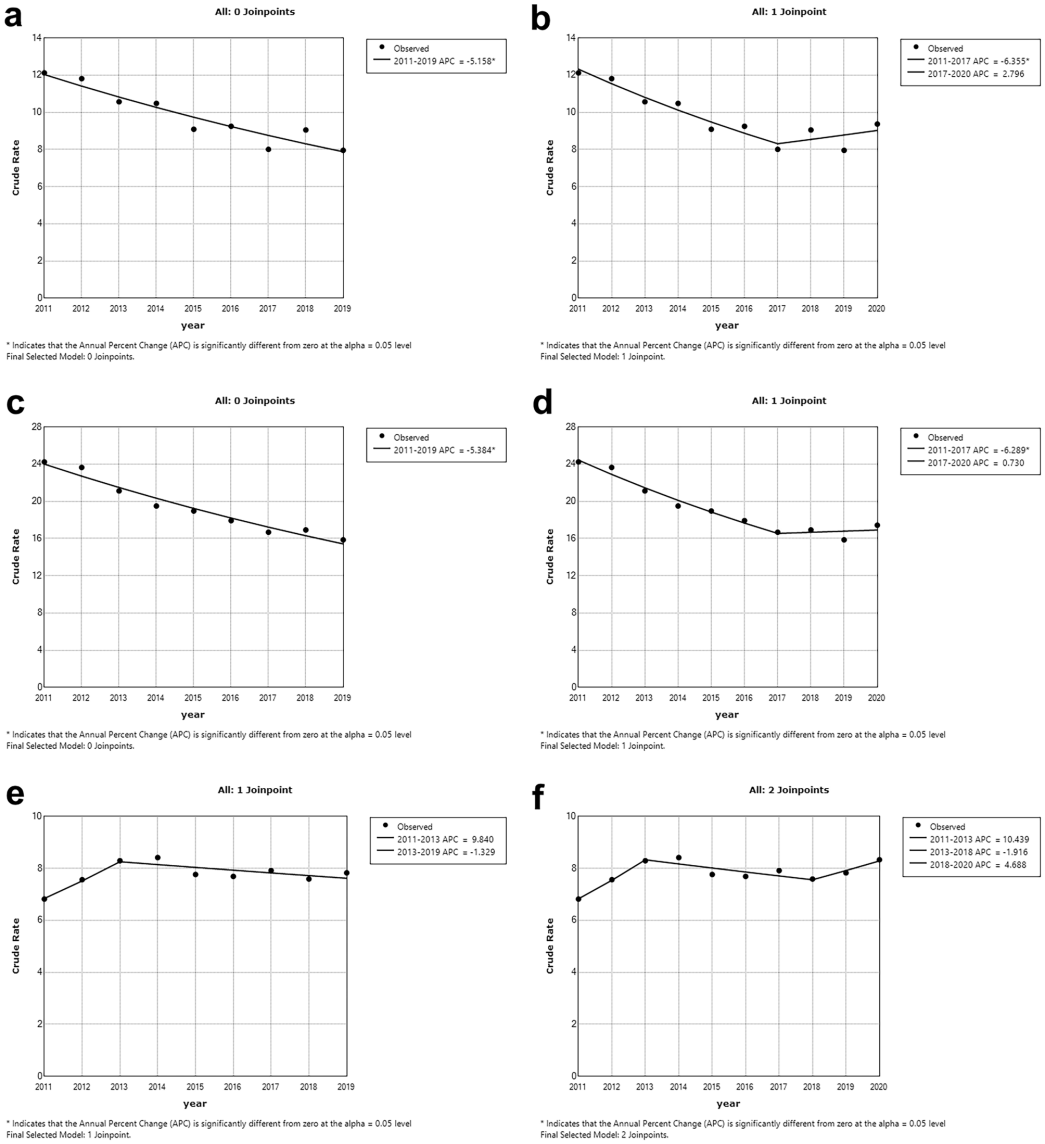
Trends in suicide death rates for countries with the highest (percentage) increase in 2020 using JP regression for the periods 2011–2019 (left side) and 2011–2020 (right side). (**a,b)** Ireland (**c,d)** Hungary (**e,f)** Spain. *Notes*: *JP* Joinpoint; *APC* Annual Percent Change.

Figure 4 shows the countries where the rise in 2020 has a trend effect: in the case of Ireland (Fig. 4a,b) and Hungary (Fig. 4c,d), a ‘purely’ downward trend was broken or reversed in 2020, with 2017 as the start of the upward phase; a similar trend can be observed in Spain (Fig. 4e,f), with a turning point in 2018, but there the decreasing trend was preceded by an ascending phase (up to and including 2013).

### The most affected countries in detail

Of countries with *significant* IRRs greater than 1, the top three (i.e., Ireland, Hungary, and Spain) were examined with a more detailed spatial breakdown (also by gender). In the case of France (ranked 4th), both male and female subpopulations showed ‘only’ single-digit increases (and were not significant), so we did not analyse this country in detail.

**Figure 5:**
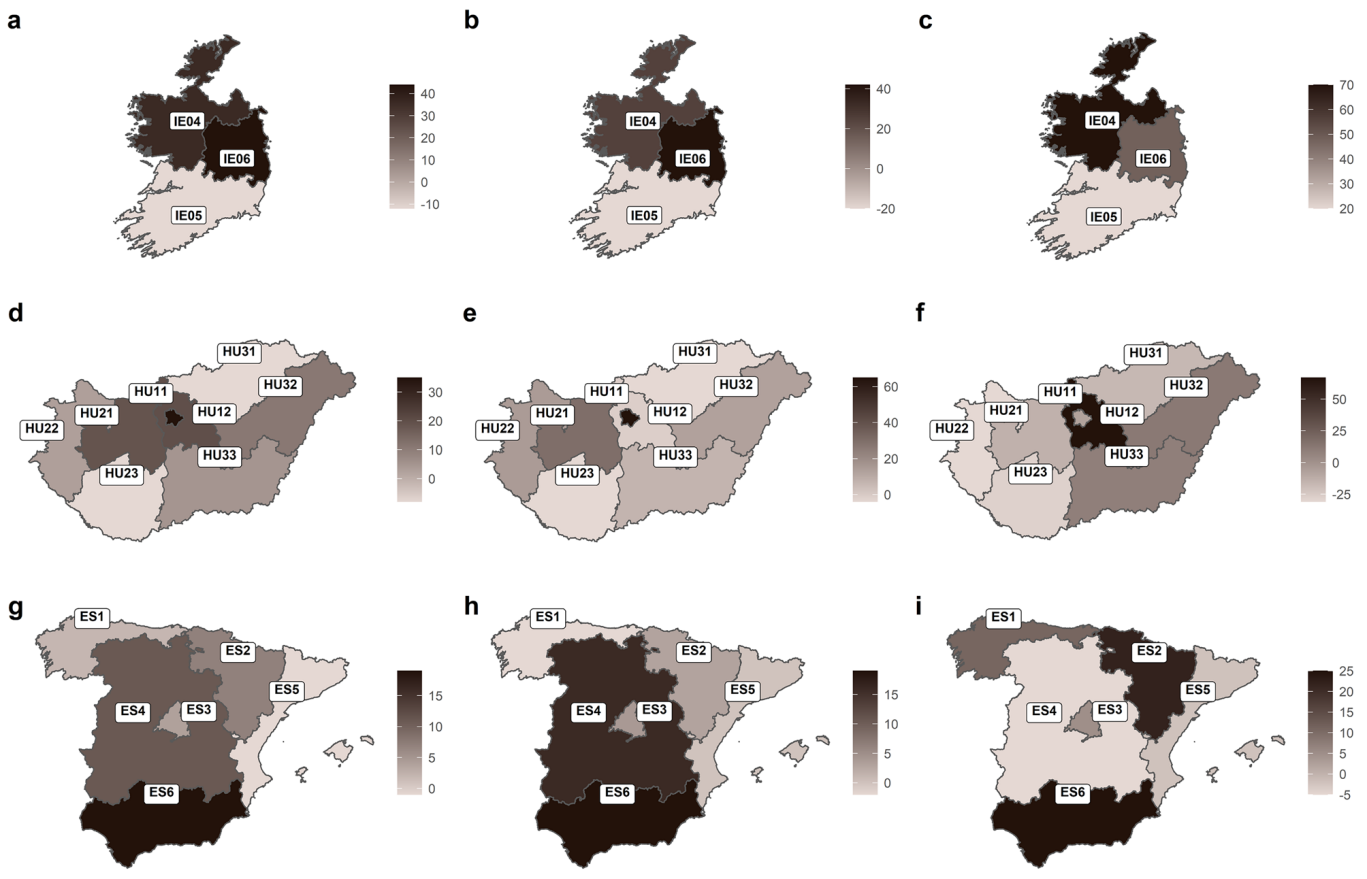
Countries most affected by the pandemic regarding annual changes in the incidence of suicide deaths. (**a–c)** Ireland (by NUTS2 level regions; whole population, male, and female subpopulation, respectively). (**d–f)** Hungary (NUTS2) (**g–i)** Spain (NUTS1). Annual changes were expressed as percentages (%). The maps were produced using the R packages *sf* (version 1.0–12; https://cran.r-project.org/web/packages/sf/index.html) and *ggplot2* (version 3.3.5; https://cran.r-project.org/web/packages/ggplot2/index.html). *Notes*: *NUTS* Nomenclature of territorial units for statistics (from the French version *Nomenclature des Unités territoriales statistiques*). Codes: Ireland, NUTS2 level: *IE04* Northern and Western Region, *IE05* Southern Region, *IE06* Eastern and Midland Region; Hungary, NUTS2 level: *HU11* Budapest *HU12* Pest County, Central Hungary, *HU21* Central Transdanubia, *HU22* Western Transdanubia, *HU23* Southern Transdanubia, *HU31* Northern Hungary, *HU32* Northern Great Plain, *HU33* Southern Great Plain; Spain, NUTS1 level: *ES1* Noroeste (Northwest), *ES2* Noreste (Northeast), *ES3* Com. de Madrid (Community of Madrid), *ES4* Centro (Centre), *ES5* Este (East), *ES6* Sur (South).

Thus, Fig. 5 shows the most affected countries in more detail (by smaller territorial units; in order of total population, then male and female subpopulations), coloured by the annual rate of change, i.e. Ireland (Fig. 5a–c), Hungary (Fig. 5d–f), and Spain (Fig. 5g–i).

In the case of Ireland, the *Southern* region (IE05) saw the most favourable change (− 12%, − 20% and + 20% overall, and for men and women, respectively), while the regions of *Eastern and Midland* (IE06) and *Northern and Western* (IE04) showed similar overall trends (+ 44% and + 36%, respectively), but differed by gender (males: + 42% vs. + 25%; females: + 48% vs. + 70%).

In Hungary, the capital city (*Budapest*, formerly part of *Central Hungary*; HU11), the surrounding region (*Pest County*, formerly part of *Central Hungary*; HU12) and its western neighbour (*Central Transdanubia*; HU21) fared the worst (+ 35%, + 20%, and + 19%, respectively). However, broken down by gender, we found a different picture here as well: for men, Budapest and Central Transdanubia were the most affected regions (+ 65% and + 30%, respectively), while the Pest region stagnated (− 1%); for women, Pest saw the highest increase (+ 67%), while both Budapest and Central Transdanubia showed a slight decrease (− 7% and − 14%, respectively).

In Spain, the largest increases were recorded in the southern (*Sur*; ES6) and central (*Centro*; ES4) regions, followed by the north-eastern (*Noreste*; ES2) region (+ 19%, + 11%, and + 7%, respectively). There was some variation by gender: for males, the regions South and Centre performed again the worst (+ 19% and + 16%, respectively), but for females, the region Northeast stood out alongside the South (+ 25% and + 22%, respectively), while the Centre was the best-performing region (− 5%). However, it should be noted that the Canary Islands (*Canarias*; ES7), the outermost and by far the least populous region, had been excluded from the above analyses and comparisons due to their very different nature.

## Discussion

Among the countries with the highest suicide rates in 2020—out of the 27 examined countries –, there are Northern European (Baltic states), Eastern European (Hungary), Western European (Belgium, France) and Southern European (Slovenia) countries, as well.

In general, the countries with the lowest suicide rates came from Southern Europe (Greece, Italy, Spain), the only real exception being Slovakia (Eastern Europe), the northern neighbouring country of Hungary. This is interesting (in itself), especially because Hungary and its southwestern neighbour, Slovenia, were among the top three countries with the highest suicide rates.

In almost two-thirds of the countries studied, suicide rates did not increase between 2019 and 2020. Ireland, Hungary, Estonia, Spain, Latvia, and France recorded the highest increases. However, Lithuania, which was ranked as the third among Baltic states, showed one of the highest decreases in suicide rates.

It should be noted that the increase observed in Estonia and Latvia was not statistically significant (due to their low population size), while in the case of France, it was just ‘significant’, but the increase in volume was not ‘negligible’.

However, for Ireland, Hungary, and Spain, the significant increase in 2020 also reversed the previous downward trend, suggesting that these three countries were the hardest hit by the pandemic in terms of suicide mortality rates. However, it is also worth mentioning that the proportion of Catholics declined considerably in these countries from 2011 to 2022 (see Supplementary Fig. S2 online) which may also have contributed to this. The direction and extent of the changes in the suicide rates varied by region in these three countries and differed significantly by gender, as well.

Overall (considering all the countries examined), there was no statistically significant change (increase) in suicide death rates for either men or women. Among the age groups, the rise was significant only in the oldest age group (aged over 65 years), but even within that exclusively in the subgroup of (some) Catholic-majority countries.

Suicide mortality can increase during infectious disease outbreaks^30^; therefore, it was hypothesised that the COVID-19 pandemic might elevate suicide mortality. However, an international study that investigated 21 higher-income countries in the early months of the pandemic reported that there was “no evidence of a significant increase in the risk of suicide since the pandemic began in any country or area”^31^. A later study by the same international collaboration, using global longitudinal data from 33 countries during the first 9–15 months of the pandemic, published similar conclusions^32^.

A preliminary Hungarian study revealed a significant increase during the first year of the pandemic^3^. A possible reason for this is the high social acceptance of suicide (still nowadays): that is, Hungarians tend to turn to suicide as a ‘solution’ in a crisis like the pandemic^33,34^. In addition, psychiatry in Hungary was particularly hard hit by the cutbacks in the healthcare sector: beds and specialists in many hospitals were removed from here at the earliest and returned here at the latest.

Our previous report detected a reversed trend in suicide mortality during the pre-vaccination period compared to the pre-pandemic period in Hungary (10 years before the pandemic)^35^. Additionally, significant increases in suicide rates were detected in the two regions with the lowest COVID mortality rates (Central Hungary, Central Transdanubia). This might be caused by the decline in the ‘bustling metropolitan life’ (catering industry, cultural events, tourism) due to the restrictions. The first cases of both infections and deaths were recorded in Central Hungary.

Spain has traditionally had a low suicide rate, but it may be undermeasured due to the high proportion of undetermined causes of death^36^. In the early stages of the pandemic, Spain was one of its epicentres, along with Italy.

Andalusia, which accounts for a large part of the population of the southern region (comprising Andalusia, Murcia, Ceuta, and Melilla), is Spain’s most populous autonomous community and is highly dependent on tourism. The first locally acquired COVID-19 cases in Spain were also found here, which might be associated with a sense of stigma and/or paranoia^37^; furthermore, Melilla and Ceuta are the two most densely populated autonomous communities. Ceuta, Andalusia, and Melilla are among the autonomous communities with the lowest GDP per capita. Nevertheless, the GDP per capita in Murcia isalso well below the national average.

Castilla-León, Castilla-La Mancha, and Extremadura in the central region have a GDP below the national average. Although these are the autonomous communities with the lowest population density, certain provinces of Castilla-León and Castilla-La Mancha were among the areas hardest hit by COVID-19 in May^38^.

Ireland’s first lockdown lasted the longest in Europe (especially in hospitality and retail^39^) and caused a staggering economic downturn^40^ and an increase in unemployment^41^. Due to the lockdowns, by December of that year, the infection rate was cumulatively the lowest in the entire EU. Furthermore, it had one of the lowest excess mortality in the world in 2020–2021 (only surpassed by Iceland and Norway in Europe^42^).

However, pandemic measures affected not only the economy but also—among others—health and religion^43^. In addition, the restrictions applied to funerals had a particularly negative impact on Irish people due to the strong funeral culture of the country^44^.

In Ireland, the central region had by far the highest COVID mortality rate (2–3 times higher than the other two regions^45^). The first infection and death were also registered here.

In addition to age being the most important risk factor for the severity and mortality of COVID-19 infection, it also predisposes individuals to psychosocial difficulties^46^. Isolation (especially in nursing and retirement homes) can result in deep loneliness and even depression^47^ as older adults had fewer options for managing their lives during the pandemic (disparities in access to or literacy in digital resources)^48^. It is also well-known that social disconnectedness can develop a range of diseases (neurocognitive, cardiovascular, autoimmune)^49^ which may aggravate their mental health. Moreover, misinformation and uncertainty can cause mass hysteria, and the elderly are particularly vulnerable^50^. Finally, the loss of loved ones (spouse, siblings) can also affect suicidality.

Consistent with the above, a markedly increased suicide risk for persons aged 65 years and older was found in Taiwan^51^, Spain^52^ and Hungary^53^. In 2020, increased suicide mortality in the elderly population was observed also in Italy, which was the first European country severely hit by the COVID-19 pandemic^54^.

Suicide rates/risk and protective factors vary across religions^55^. All Christian religions (Catholic, Orthodox, Protestant) consider suicide a sin. However, protestant churches allow more space for individual thinking. In contrast to Catholic churches, Protestant religions are characterised by private confession and absolution. According to Durkheim, “the only essential difference between Catholicism and Protestantism is that the latter permits free inquiry to a far greater degree than the first”^56^.

Following the above, suicide is significantly more common in Protestant populations^57^. Moreover, “although religious networks do mitigate suicides among Protestants, the influence of church attendance is more dominant among Catholics”^58^ and “deeper involvement in the church community decreases suicide risk for Catholics, but increases it for Protestants”^59^.

Religion primarily plays a role in the elderly (“older persons are more religious”) and in whom the increase in suicide rates was observed, especially among Catholics. In Hungary, Ireland, and Spain the proportion of Catholics decreased markedly between two censuses (2011, 2022) and the relative suicide mortality significantly increased during the ‘pre-vaccination period’. This might indicate the lack of the Catholic religion’s protective role in a global crisis like the COVID-19 pandemic.

To our knowledge, this is the first study to analyse the trends of suicide in the EU and related countries concerning the COVID-19 pandemic by gender, age group, and religion. The longest study period was used in direct comparison analyses.

Although we compared entire years out of necessity (due to lack of monthly data), with monthly data, it would also be possible to consider only the period affected by Covid (March–December) in the yearly comparisons.

It also raises the question of the appropriateness of picking a single year (i.e., the previous year, 2019) and using it as a basis when judging the number of suicidal deaths in 2020. In this case, it is customary to take a multi-year average (generally, 3–5 years) as a base and compare the indicators of the given year with it. However, if there is otherwise a clear (upward or downward) trend in the background, this ‘smoothing’ would lead to misleading results.

A possible ‘balancing’ of the above could be to compare the observed values of the period burdened with Covid (March–December 2020) with the hypothetically ‘expected’ values of the period that would have been predicted based on the trend of the previous, longer period (so-called ‘interrupted time-series’ method). However, the ‘trend stability’ in the (pre-)period is crucial in these analyses; unfortunately, in many cases, this has not been verified or even fulfilled.

The public mortality data of ESTAT have a long lead time of three years; that is, there have been no data available for suicide deaths (at least certified by Eurostat) in the respective countries during 2021–2023. Consequently, we have only been able to investigate suicide deaths until December 2020, which can be regarded as the ‘pre-vaccination period’ since the vaccinations started in the EU (officially) on December 27, 2020. The relatively short period of observation from the onset of the COVID-10 pandemic suggests caution in interpreting results in terms of the effect of the pandemic on suicide mortality. Previous studies focusing on the effect of catastrophes or epidemics have shown that after an initial decrease in suicide mortality, an increase is often observed.

The possibility of ecological fallacy is another limitation of our analysis: individual-level associations may not always be reflected in ecological-level associations.

Identifying the groups most at risk of the pandemic and crisis (both in economic and social terms) would be extremely important. However, the results obtained in the ecological study (at the population level) should be investigated in (more) detail in future studies; this could lead to a better understanding of the underlying causes and preventive measures.

Several countries (i.e., Cyprus, Iceland, Liechtenstein, Luxembourg, Malta, Türkiye, and the UK) were excluded from the analysis due to the lack of validated data or small population size. These would also be worth examining (even separately), but for the sake of consistency (in the data source) and statistical meaningfulness (interpretability), they are not included in this investigation.

For similar reasons, the groups of ‘youth’ (aged under 20 years) and ‘young adults’ (20–34 years) were not analysed separately: those under 35 years of age were analysed together (as one group). However, it would be worth looking at them separately, in addition to children (under 15 years of age).

To explore the heterogeneity in the trend of suicide rates, we originally used the United Nations (UN) geoscheme for Europe (i.e., Western Europe, Eastern Europe, Northern Europe, and Southern Europe), but this was not ‘suitable’ for this, despite it appearing to be the most reasonable. Religion demonstrates cultural embeddedness and, as such, may play an explanatory role. Unfortunately, (census) data on religious affiliation are very limited.

Other variables (e.g., gross domestic product [GDP], development index, globalisation index, prosperity index, happiness index, stringency index, economic support index, etc.) are also conceivable, but they do not lead to results in this regard, either. The role of additional variables (stress at the social level, social disintegration/fragmentation, territorial, and cultural diversity) is also possible, but their appropriate quantification is essential for such analyses.

What is needed is a (validated) indicator that quantifies the accessibility of health care and in particular, psychiatric care; this would make it possible to compare countries concerning an explanatory variable that could potentially greatly influence the increase in the number of suicides or the lack thereof.

It is worth mentioning that there is a variability in the quality of cause-of-death data across the European countries that might affect comparisons of suicide mortality. Due to differences in registration or data processing procedures, the number of misclassified or underreported suicides from death certificates might vary significantly from one country to another.

Overall, there was no statistically significant increase in suicide death rates in Europe considering all the countries examined. However, the pattern of suicide rates has changed significantly in some countries. In addition to the change in the regional pattern of suicide rate, the increase was significant in the oldest age group, over 65 which was even more marked within some Catholic-majority countries (Hungary, Ireland, and Spain) during the ‘pre-vaccination period’; in these countries, a noticeable decline in the proportion of Catholics was recorded.

It became clear early that the virus was primarily a threat to the older population (over 65 years of age), and those with chronic diseases (which are more prevalent among the elderly). During the pandemic (and especially during the closures), the quality of social relations remained far below expectations for a long time, leading to loneliness in many mentally vulnerable people. Therefore, the development of social safety nets, also at a state level, should be a priority.

To our knowledge, our work was the first cross-national ecological study to reveal these findings. A more precise examination of the results could be carried out based on individual anamnestic histories.

## Methods

### Study population and suicide data

Data on the population and number of deaths were obtained from the *Data Browser* published online by the European Statistical Office, Eurostat (*ESTAT*^45^). Mortality data were classified according to the 10th revision of the International Classification of Diseases (ICD-10); codes concerning ‘intentional self-harm’ were X60-X84 and Y87.0 (‘sequelae of intentional self-harm’). The mid-year population was calculated as the average start-of-the-year population recorded over two consecutive years.

Both the population and number of suicide deaths were initially broken down by age group as follows: 0–19 (‘youth’), 20–34 (‘young adults’), 35–49 (‘middle-aged adults’), 50–64 (‘older adults’) and over 65 years (‘elderly’/pensioners). However, due to the lower number of cases in the under-20 age group, the first two groups were analysed together.

Suicide mortality data were available only for 32 of the 34 countries included in the ESTAT database for the years 2019 and 2020 (with the exceptions being the UK [United Kingdom] and Türkiye [formerly Turkey]). However, the two microstates (Liechtenstein, Malta) and ‘less populous’ countries (Cyprus, Iceland, and Luxembourg) were excluded from the analysis due to the relatively low number of cases.

Consequently, 27 countries were included in the analysis: 24 current member states of the European Union (*EU*), one EU candidate country (Serbia), and two member countries (Norway, Switzerland) of the European Free Trade Association (*EFTA*).

Territorial units smaller than countries were based on the first/second level of NUTS 2021 classification (Nomenclature of Territorial Units for Statistics [abbreviated from the French version *Nomenclature des unités territoriales statistiques*], 2021 revision^60^).

### Statistical analyses

Incidence rate ratios (IRRs) and their 95% confidence intervals (95% CIs) were originally calculated to characterise annual changes (from 2019 to 2020) in the incidence of suicide deaths. These effect sizes were then pooled using meta-analytic techniques (employing a random-effects model with the generic inverse variance method).

Statistical heterogeneity was also measured by applying the chi-square Q test (threshold *p* = 0.1) and quantified by using the I-squared index (I^2^). The latter is categorised at 25%, 50%, and 75% as low, moderate, and high heterogeneity, respectively.

Finally, sensitivity and subgroup analyses were performed to explore the heterogeneity in the effect size across countries. These analyses were based on the religious distribution of countries, as follows: Catholic-majority, Protestant-majority, mixed Catholic-Protestant and Orthodox-majority countries, respectively.

The investigations mentioned above were carried out overall and then separately by gender and age group. The results were displayed on forest plots. All calculations and figures were performed using R (version 4.3.0; R Core Team 2023).

Additionally, to decide whether the Covid period (and the possible increase in the suicide rate) was accompanied by a reversal of the downward trend in suicide rates, we applied the joinpoint (JP) regression model (using Poisson variance, the permutation test and a maximum of two joinpoints); related plots were generated by the Joinpoint Regression Program (version 4.9.1.0; National Cancer Institute 2022).

### Supplementary Information

Supplementary Information.

## Data Availability

The data used in this study are available from the Data Browser published online by the European Statistical Office, Eurostat (ESTAT), https://ec.europa.eu/eurostat/databrowser/explore/all/all_themes.

## Abbreviations

ESTAT: European Statistical Office
ICD: International Classification of Diseases
NUTS: Nomenclature of Territorial Units for Statistics (from the French version Nomenclature des Unités Territoriales Statistiques)
IRR: Incidence rate ratio
CI: Confidence interval
ASMR: Age-standardised mortality rate
JP: Joinpoint
APC: Annual percent change
