# Supplementary material for: The impact of the first year of COVID-19 pandemic on suicides in a collection of 27 EU-related countries
Source: Sci Rep. 2024 Jul 30;14:17671. doi: 10.1038/s41598-024-68604-3 (PMC11291984; doi:10.1038/s41598-024-68604-3)
Supplement: Supplementary file 1 — Supplementary Information. [file 41598_2024_68604_MOESM1_ESM.pdf]

Supplementary Information

THE IMPACT OF THE FIRST YEAR OF COVID-19 PANDEMIC ON SUICIDES IN A COLLECTION OF 27 EU-RELATED COUNTRIES

Tamás Lantos [1, *, ID], Tibor András Nyári

[ID] ORCID ID: 0000-0002-5081-7313

[*] Correspondence: lantos.tamas@med.u-szeged.hu

[1] Department of Medical Physics and Informatics, Albert Szent-Györgyi Medical School, University of Szeged, 9 Korányi alley, 6720, Szeged, Hungary

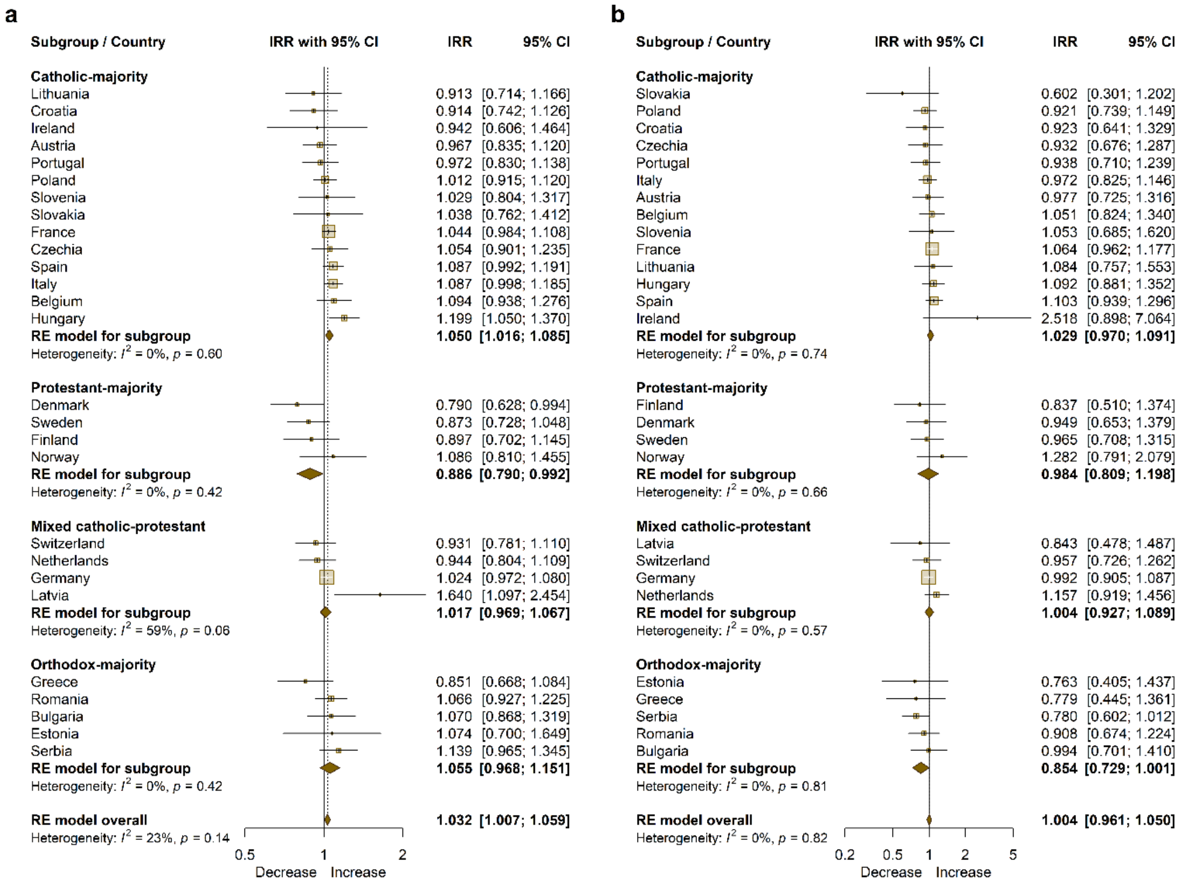

Fig. S1. Forest plots for subgroup analyses of annual changes in the incidence of suicide deaths. **a.** Male subpopulation aged over 65 years. **b.** Female subpopulation aged over 65 years. The plots were generated using the R *meta* package (version 6.1-0; https://cran.r-project.org/web/packages/meta/index.html).

Changes in the proportion of Catholics

While census data on religious affiliation were available in Hungary and Ireland [1, 2], in Spain only the results of sociological surveys characterise the religious distribution [3]. In all three countries, Catholicism is the most widespread religion.

Examining the available data for years 2011 and 2022, the number of Catholics in relation to the total population decreased markedly (**Fig. S2**): from 84.2% to 68.8% in Ireland, from 73% to 56.2% in Spain, and from 39% to 30.1% in Hungary (resulting in relative decreases of the proportions [compared to the base year 2011]: 18%, 23% and 23%, respectively).

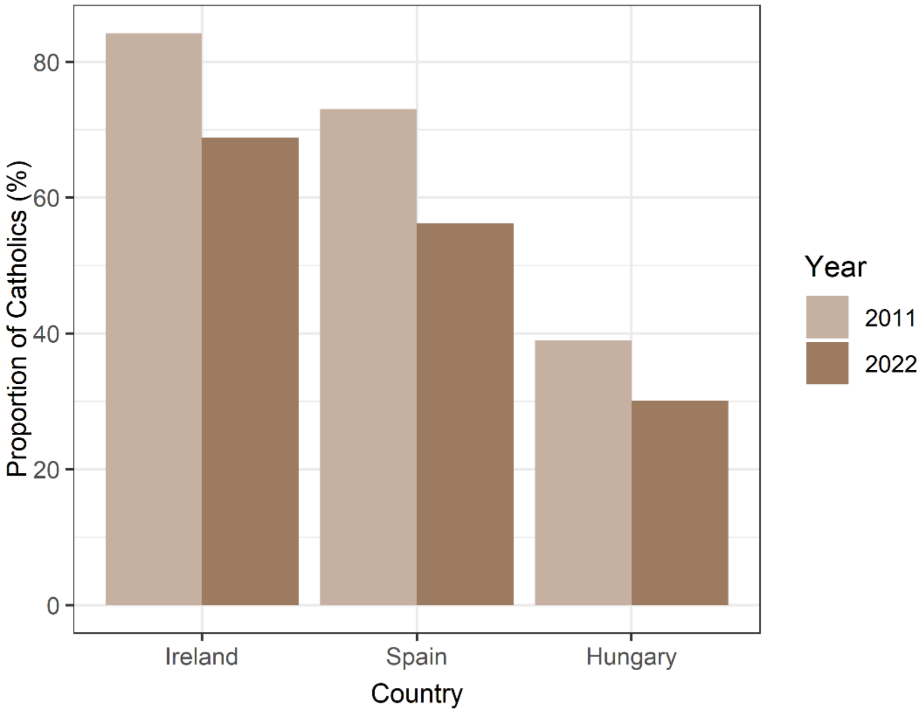

Fig. S2. Share of the population who consider themselves Catholic in 2011 and 2022.

References

1. Hungarian Central Statistical Office. *Census 2022: Population by ethnic attributes, county and type of settlement.* https://nepszamlalas2022.ksh.hu/en/database/#/table/WBS003/ (2023).

2. Central Statistics Office (Ireland). *Census 2022: Diversity, Migration, Ethnicity, Irish Travellers & Religion.* https://data.cso.ie/table/F5135 (2023).

3. Centre for Sociological Research (Spain). *Monthly barometers 2011-2022.* https://www.cis.es/catalogo-estudios/resultados-definidos/barometros (2022).
